# P-732. Clinical Outcomes in Adults Hospitalized with Viral Lower Respiratory Tract Disease with a Bacterial and/or Fungal Coinfection: A US Claims Data Base Study

**DOI:** 10.1093/ofid/ofae631.928

**Published:** 2025-01-29

**Authors:** Susan J Johnson, Ekaterina Maslova, Malin Fageras, Gopal Dalal, Hashmath Ulla T A Syed, Nadir Yehya

**Affiliations:** AstraZeneca, Cambridge, England, United Kingdom; AstraZeneca, Cambridge, England, United Kingdom; AstraZeneca, Cambridge, England, United Kingdom; ZS Associates India Pvt. Ltd., Bengaluru, Karnataka, India; ZS Associates India Pvt. Ltd., Bengaluru, Karnataka, India; Children’s Hospital of Philadelphia, Philadelphia, Pennsylvania

## Abstract

**Background:**

Bacterial and fungal coinfections in people hospitalized for viral lower respiratory tract disease (LRTD) may worsen clinical outcomes. We describe US adults hospitalized with viral LRTD and diagnosed with bacterial/fungal coinfection in a large US claims database.

**Table 1:**
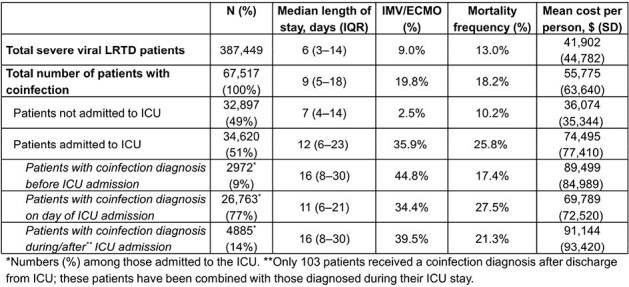
Clinical outcomes in patients with severe viral LRTD diagnosed with a bacterial or fungal coinfection

**Methods:**

We included adults with a first hospitalization for viral LRTD (2015–2023) based on ICD codes using Optum’s de-identified Clinformatics® Data Mart Database. From this cohort, we identified patients with a bacterial or fungal coinfection using ICD codes. We descriptively analysed patient characteristics in the 12 months pre-hospitalization and clinical outcomes for the first 30 days from hospital admission.

**Results:**

We identified 387,449 patients with severe viral LRTD (75% with coronavirus etiology), of which 67,517 (17%) also had a bacterial/fungal coinfection diagnosis. Mean (SD) age was 73 (14) years in these patients and 54% were women (similar to the overall viral LRTD cohort). Mean (SD) Charlson Comorbidity Index (CCI) was 4 (3), which was slightly higher than in the overall cohort (mean CCI [SD]: 3 [3]). Just over half (51%) had an intensive care unit (ICU) admission (overall cohort: 39%), and among these patients 91% received their coinfection diagnosis while in the ICU (Table). Outcomes including invasive mechanical ventilation (IMV)/extracorporeal membrane oxygenation (ECMO) and death were more frequent in patients with coinfections versus the overall cohort. Among patients receiving IMV/ECMO, 73% received their coinfection diagnosis on the day of the procedure (12% were diagnosed before; 15% were diagnosed after receiving IMV/ECMO). Length of stay was shorter among those diagnosed with a coinfection at ICU admission (vs those diagnosed before ICU admission or during/after an ICU stay). IMV/ECMO frequency was highest in those who were diagnosed with coinfection before their ICU admission; mortality was higher among those diagnosed during their ICU stay.

**Conclusion:**

Patients with viral LRTD and bacterial/fungal coinfection had a higher frequency of severe clinical outcomes compared with the overall viral LRTD cohort, that could not be fully explained by a high ICU admission in this cohort. Coinfection may be an important consideration for high-risk patients even prior to ICU admission.

**Disclosures:**

**Susan J. Johnson, PhD**, AstraZeneca: Employee|AstraZeneca: Stocks/Bonds (Public Company) **Ekaterina Maslova, ScD**, AstraZeneca: Employee of AstraZeneca|AstraZeneca: Stocks/Bonds (Private Company) **Malin Fageras, PhD**, AstraZeneca: Full time employee|AstraZeneca: Stocks/Bonds (Private Company) **Gopal Dalal, PhD**, AstraZeneca: Advisor/Consultant|AstraZeneca: Employee of ZS Associates India Pvt. Ltd. and contracted to AstraZeneca at time of study **Hashmath Ulla T. A. Syed, PhD**, AstraZeneca: Advisor/Consultant|AstraZeneca: Employee of ZS Associates India Pvt. Ltd. and contracted to AstraZeneca at time of study **Nadir Yehya, MD**, AstraZeneca: Advisor/Consultant

